# Retargeting T Cells for HER2-Positive Tumor Killing by a Bispecific Fv-Fc Antibody

**DOI:** 10.1371/journal.pone.0075589

**Published:** 2013-09-23

**Authors:** Lei Wang, Yanran He, Ge Zhang, Juan Ma, Changzhen Liu, Wen He, Wei Wang, Huamin Han, Bhargavi M. Boruah, Bin Gao

**Affiliations:** 1 Chinese Academy of Sciences Key Laboratory of Pathogenic Microbiology and Immunology (CASPMI), Institute of Microbiology, Chinese Academy of Sciences, Beijing, China; 2 University of Chinese Academy of Sciences, Beijing, China; 3 University of Chinese Agriculture, Beijing, China; 4 Hebei Key Laboratory of Medical Biotechnology, Hebei Medical University, Shijiazhuang, China; 5 China-Japan Joint Laboratory of Molecular Immunology and Microbiology, Institute of Microbiology, Chinese Academy of Sciences, Beijing, China; University of Pécs Medical School, Hungary

## Abstract

To exploit the biological and pharmacological properties of immunoglobulin constant domain Fc fragment and increase the killing efficacy of T cells, a single chain variable fragment specific to CD3 was fused with Fcab (Fc antigen binding), a mutant Fc fragment with specificity against Human epidermal growth factor receptor 2 (HER2) developed by F-star. The bispecific fusion named as FcabCD3 was expressed by transient transfection in HEK-293T cells and purified by affinity chromatography. Specific cytolytic activity of retargeted T cells to kill HER2 positive SKBR3 cell line was evaluated *in vitro*. FcabCD3 was able to retarget T cells to kill both Herceptin insensitive Colo205-luc cell line and HER2 low expression MDA-MB-231-luc cell line. Furthermore, FcabCD3 was effective in eliminating the Colo205 tumor established on BALB/c nu/nu mice.

## Introduction

Antibodies have emerged as one of effective therapeutics against a plethora of diseases including cancer and auto-immune disorders [1,2]. Antibodies possess high affinity and stringent specificity to target antigens while rendering the targeted partner susceptible to antibody-dependent cell-mediated phagocytosis (ADCP) and antibody-dependent cell-mediated cytotoxicity (ADCC) [3]. To expand the application of antibody-based therapeutics, antibodies have been further designed to bind simultaneously to two different antigens. Such antibodies with bispecific or multispecific targeting abilities have offered wide applications in both diagnostics and therapeutics. Until date, the majority of bispecific antibodies in the clinic include: 1, Simultaneous targeting of multiple receptors; 2, Delivery of payloads, toxins and other functional proteins [4,5]; 3, Recruitment of effector cells through one of FcγR receptors or CD3 receptor to tumors with a tumor associated marker [6]. The most successful bispecific antibodies (bsAb) belong to those that recruit cytotoxic T cells to the site of tumor cells, for instance, Catumaxomab, an anti-EpCAM/CD3 bispecific antibody and Blinatumomab, an anti-CD19/CD3 bispecific antibody. The approval of Catumaxomab (anti-EpCAM/CD3) by the European Union (EU) highlighted the potentials of bsAb as attractive candidates for antibody-based immunotherapy.

Classical methods to produce bispecific antibodies include hybrid-hybridoma (quadroma) and chemical cross-linking. DNA recombinant technology has led to the development of various formats of new bispecific antibody (bsAb) during the last decades, including tandem single chain variable fragments, diabodies, single chain diabodies, dock-and-lock trivalent Fabs, heterodimeric ScFvs or Fabs, dual-affinity re-targeting (DART) molecules [7,8], as well as derivatives thereof. However, all these formats lack Fc fragment comparing to conventional antibody that has been demonstrated to be very successful as therapeutic agents. Fc domain has been demonstrated to markedly increase antibody’s plasma half-life by interaction with the salvage neonatal Fc-receptor [9], as well as to the slower renal clearance [10], which are critical factors for the maintenance of therapeutic activity. The importance of the interaction between Fc domain and FcR was reflected in the experiments in which antibodies lost antitumor therapeutic activity when administrated into common γ chain knockout mice lacking the activating FcR [11,12]. As such, it would be desirable to produce a bispecific antibody format with an Fc fragment that matches a conventional IgG antibody. 

Human epidermal growth factor receptor 2 (HER2) is a trans-membrane tyrosine kinase receptor over-expressed in 25~30% of breast cancers [13,14]. It has served as a target for various antitumor therapies. Trastuzumab (Herceptin, Genentech), a recombinant monoclonal antibody against HER2, specifically binds to the extracellular portion of HER2, inhibits the proliferation of human cancer cells and becomes an important therapeutic option for patients with HER2 positive breast cancer. So far, anti tumor effect of Herceptin is limited to HER2 high expressers or HER2-amplified cases [15]. Bispecific antibodies (bsAb) as one of the promising treatment methods become a new choice for HER2 positive cancer immunotherapy. 

In this study, to exploit biological and pharmacological properties of immunoglobulin constant domain Fc fragment and increase killing efficacy of T cells, we constructed a bispecific antibody format by fusing a CD3 binder to Fcab, a mutant Fc fragment with specificity against Human epidermal growth factor receptor 2 (HER2) developed by F-star [16]. The new bsAb format FcabCD3 could direct T cells to target HER2 positive tumors both *in vitro* and *in vivo* while retaining the benefits of Fc fragment including longer half-life and better pharmacokinetics. The format could be used to construct a novel class of bispecific antibodies for cancer immunotherapy.

## Materials and Methods

### Ethics statement

All animals were cared for and maintained under the supervision and guidelines of the Institutional Ethic Committee of the Institute of Microbiology, Chinese Academy of Sciences (permit number PZIMCAS2012005). All surgery was performed under sodium pentobarbital anesthesia by the principle of minimize animal suffering.

The approval for using human PBMCs was obtained from Institute of Microbiology, Chinese Academy of Sciences, Research Ethics Committee (permit number PZIMCAS2012006). The anonymous blood was obtained from Beijing Red Cross Blood Centre.

### Cell Culture and Preparation of Activated T Cells

Human colorectal adenocarcinoma cell line Colo205-luc, human leukaemic cell line K562-luc, murine melanoma cell line B16F10-luc and human breast carcinoma cell lines MDA-MB-231-luc expressing the luciferase (luc) reporter gene were purchased from Caliper Life Sciences, PerkinElmer. SKBR3 and HEK-293T cell lines were originally from ATCC and maintained in our lab. Colo205-luc, K562-luc and SKBR3 were cultured in RPMI1640 (Invitrogen) supplemented with 10% FCS (fetal calf serum, Hyclone) while MDA-MB-231-luc, B16F10-luc and HEK-293T were grown in DMEM (Invitrogen) supplemented with 10% FCS at 37°C in a 5% CO_2_ humidified incubator. Activated T cells (ATCs) from normal donors were expanded *in vitro*. PBMCs were isolated from heparinized blood of healthy human donors by Ficoll (Invitrogen) density centrifugation. The PBMCs were stimulated using 10 µg/ml OKT3 (Ortho Pharmaceutical Corporation) for 3 days in RPMI-1640 medium supplemented with 10% FBS and expanded for 14 days in the presence of 100 IU/ml interleukin 2 (R&D Systems) for 14 days. FACS analysis showed that more than 98% of the remaining cells were CD3 positive T cells, these T cells were later used as effector cells.

### Construction of the plasmids

**Figure 1:**
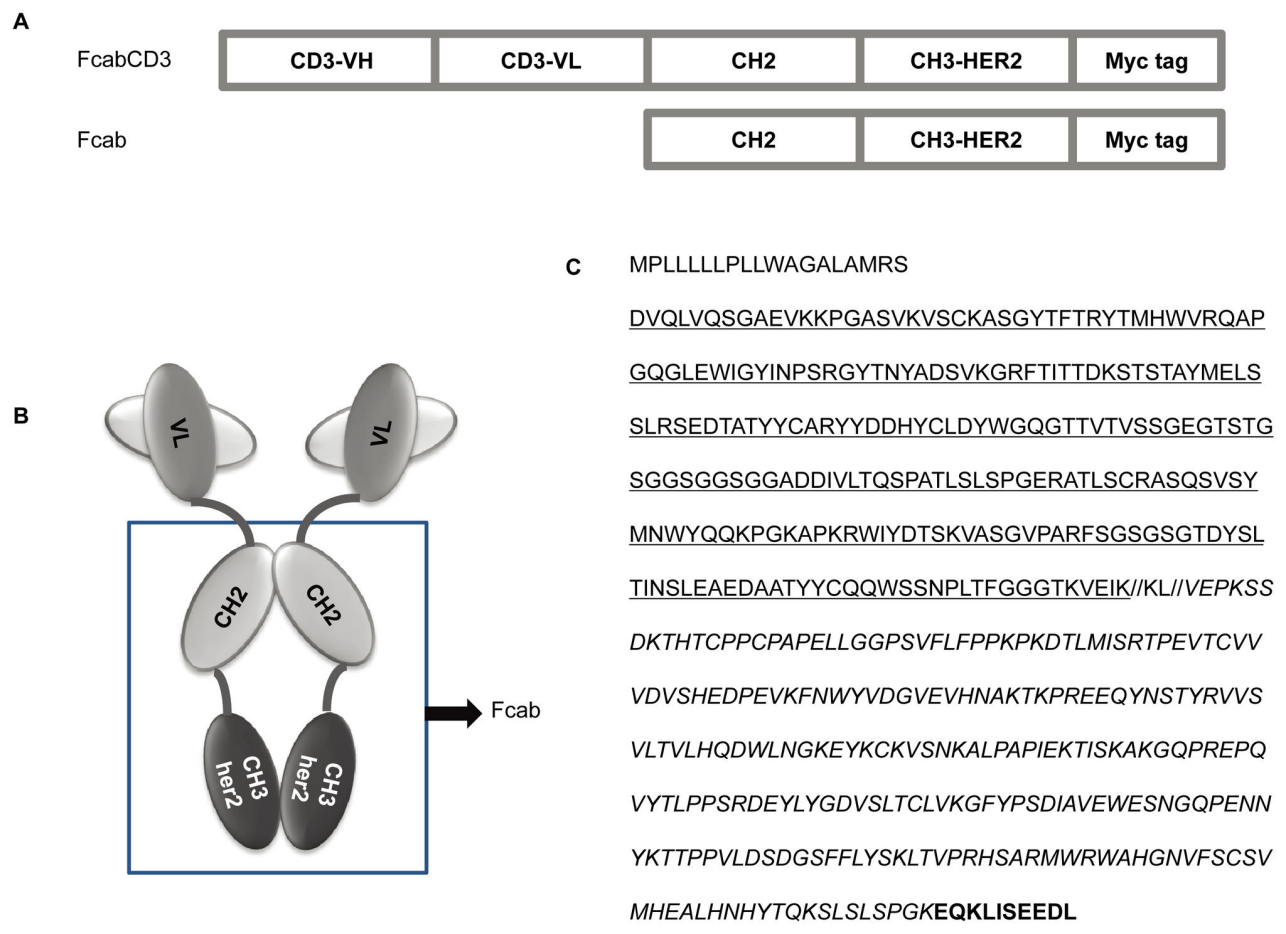
Construction and expression of a novel bispecific antibody: FcabCD3. A, Diagram of PDC316-FcabCD3 and its parent HER2 specific Fc fragment (Fcab) used in this study;. B, Schematic representative models for FcabCD3 and Fcab. The mutated CH 3 region specifically bound to HER2 antigen on the tumor cells, anti CD3 binder was inserted into the upstream of the Fcab;. C, Sequence of FcabCD3. CD33 signal peptide (The first line); anti-CD3 binder (underline); Fcab (Italic); myc tag (bold); KL: linker.

The plasmid for expression of pDC316-FcabCD3 was constructed according to the map shown in Figure 1. cDNA for Fc fragment with engineered HER2 binding site (Fcab) [16] and a CD3 binder targeting human CD3 (wo2005/040220) were synthesized by GeneRay (Beijing, China) company. Fcab was synthesized between Hind III and Sal I, the cDNA encoding CD3 binder was inserted into pDC316 plasmid flanked by the restriction sites Bgl II and Hind III, CD33 signal peptide (MPLLLLLPLLWAGALA) was inserted into the pDC316 plasmid between EcoR I and Bgl II. The same strategy was applied to the Fcab only cassette.

### Expression of FcabCD3 and Fcab

Both FcabCD3 and Fcab were expressed through transient transfection of HEK-293T by calcium phosphate precipitation method. 6 hours later, the transfection reagent was removed and the medium was replaced with fresh DMEM containing 1 mg/ml Bovine Serum Albumin or BSA (FCS was avoided for ease in further steps of protein purification). After 48 hours, the supernatants were collected, centrifuged, ﬁltered and pH was adjusted to 7.2 with 1M NaOH prior to purification. Antibodies were purified from the supernatants by affinity chromatography using Protein-A column following the manufacturer’s protocol (GE). 

**Figure 2:**
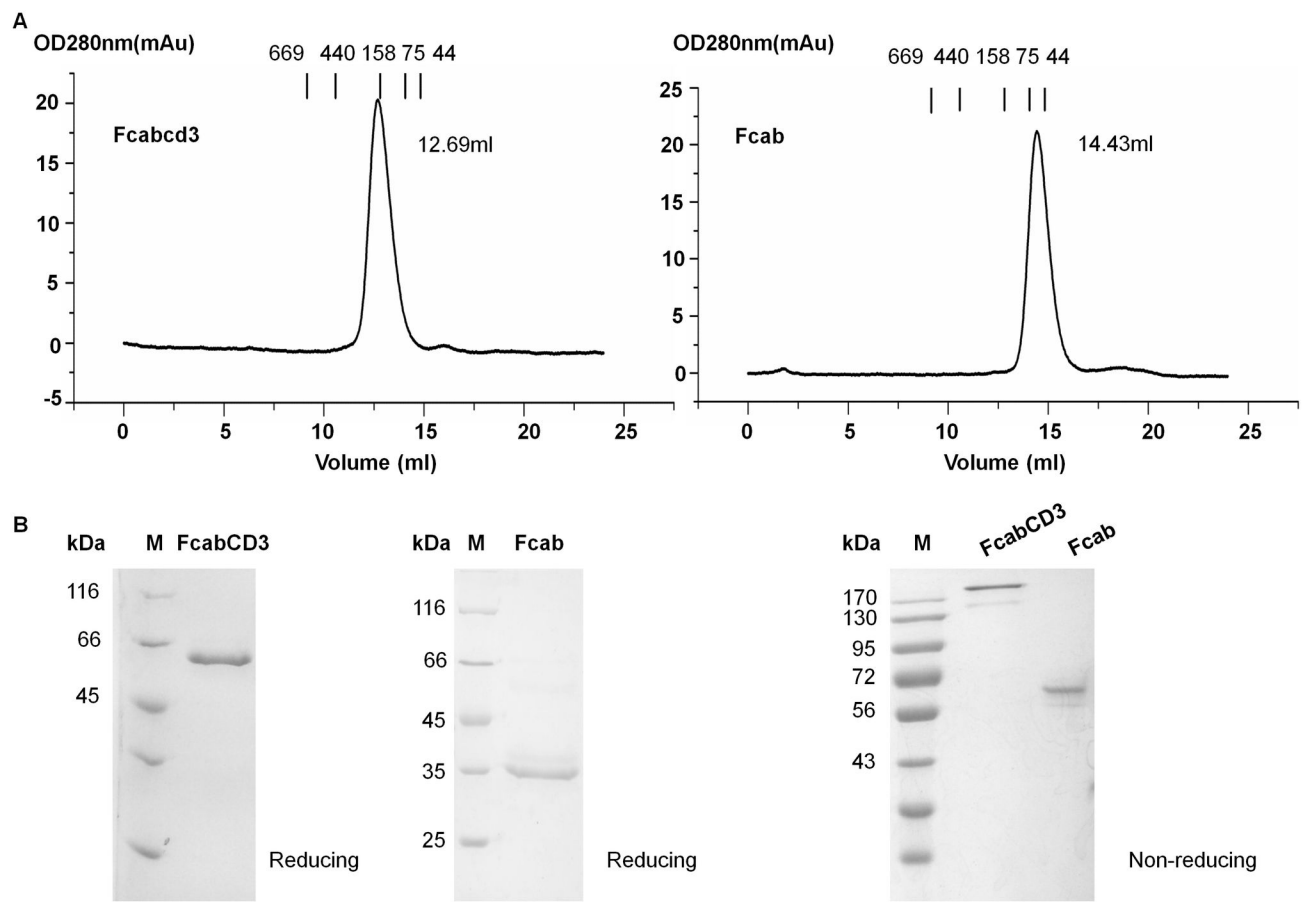
The expression and analysis of FcabCD3 and Fcab molecules. A, Purified FcabCD3 and Fcab proteins were analyzed by size exclusion chromatography with a Superdex 200^TM^ column. The makers were labeled on the top of the curves;. B, SDS-PAGE analysis of Fcabcd3 and Fcab under both reducing and non-reducing conditions on 12% SDS-PAGE gels.

Both FcabCD3 and Fcab were assessed by size exclusion chromatography using a Superdex 200^TM^ (GE Healthcare) column on AKTA purifier 2000 system according to the manufacturer’s instructions. Size exclusion chromatography was carried out in PBS at a flow rate of 0.5 ml/min. Molecular weight was determined by Gel Filtration Calibration Kit HMW (high molecular weight, GE healthcare). The makers were labeled on the top of the curves (Figure 2A). Purified proteins were analyzed on 12% SDS-PAGE under reducing and non-reducing conditions (Figure 2B).

### FACS analysis

1x10^5^ cells of each cell line, SKBR3, Colo205-luc, MDA-MB-231-luc , K562-luc, B16F10-luc and T cells in a staining buffer (PBS containing 0.5% w/v BSA and 2 mM EDTA, pH7.2) were incubated with serial dilutions of FcabCD3 or Herceptin on ice for 1 hour. The samples were washed twice with staining buffer to remove the excess antibodies. Cells were then incubated with a secondary FITC-labeled goat anti-human antibody (CWbio, Beijing) on ice for half an hour. The samples were then washed twice with staining buffer. Data for individual samples were acquired on the Guava Easycyte Mini (Guava Easycyte) and analyzed with FlowJo software.

### FcabCD3-directed cellular cytotoxicity to human tumor cell lines

Activated T cells (ATCs) were prepared as mentioned above. Specific cytotoxicity of FcabCD3 armed ATCs against HER2-expressing SKBR3 cells was evaluated using Cell Counting Kit 8 (CCK8) assay [17]. In brief, 1x10^4^ SKBR3 cells were seeded in a flat-bottom 96-well plate overnight to allow cells to adhere. On the sencond day, cryopreserved ATCs were thawed, washed, counted, and incubated with indicated doses of FcabCD3 for half an hour at room temperature. Then FcabCD3 armed ATCs were added into 96-well plate and incubated with SKBR3 cells for 2 days. After the incubation period, non-adherent cells were washed away with PBS three times. 100 µl culture medium containing 10% Cell Counting Kit 8 (CCK-8, Dojindo Molecular Technologies Inc.) was added into each well according to manufacturer’s protocol. Absorbance at OD_450_ nm was detected by BioTek plate reader (BioTek instruments) and the percentage of dead cells was calculated as supplier’s protocol.

Except for SKBR3 cell line, all the other cytotoxicity assays (against Colo205-luc, K562-luc, MDA-MB-231-luc, B16F10-luc) were performed based on bioluminescent assay [18]. The procedure was similar to the SKBR3 killing assay except that the incubation time was 20 hour. 

The cells were gently washed once with PBS, and then treated with 50 µl lysis buffer (25 mM Glycylglycine, 2 mM EGTA, 15 mM MgSO_4_, 1% Triton X-100, pH 7.8) and incubated on ice for 15 min. After three freeze-thaw cycles, the cells were centrifuged at 2500 rpm for 2 min at 4°C and supernatants were transferred into a white opaque 96-well plate pre-chilled on ice. Substrate buffer (1 M HEPES, 5 mM D-Luciferin Potassium Salt, 3 mM ATP, 15 mM MgSO_4_) was diluted with Assay buffer (2 mM ATP, 25 mM Glycylglycine pH7.8, 15 mM MgSO_4_) in the ratio of 1:20. 50 µl of this luciferin substrate solution was added into each well automatically and the resulting light was detected immediately using Synergy TM H4 Multi-Mode Microplate Reader (Synergy BioTek) at room temperature.

The specific killing was calculated using the following formula: (C-S)/(C-O)×100%. C is the luminescence value of the control group with un-armed ATCs and the target cell line. S is the luminescence value of the sample containing FcabCD3 armed ATCs and the target cell line O is the luminescence value of the medium only. All assays were performed in triplicate.

### Blocking of FcabCD3 killing by Herceptin

Competitive binding analysis of FcabCD3 with Herceptin was performed on Colo205-luc cells. In brief, T cells were pre-incubated with different concentrations of Herceptin (5 fold serial dilutions starting from 10 µg/ml) for 20 min, and then incubated with FcabCD3 (100 ng/ml) for another 10 min. 1×10^4^ Colo205-luc cells were mixed with ATCs (at E:T ratio10) for 20 hour. The cytotoxicity assay was performed following the method base on bioluminescent assay.

### Colo205-luc xenograft tumor model with BALB/C nu/nu mice

7 to 8 weeks old BALB/C nu/nu mice were purchased from VitalRiver (Beijing, P. R. China). Colo205-luc cells were harvested, counted and resuspended in PBS to a final concentration of 1x10^7^ cells/ml. Colo205-luc cells in 0.1 ml PBS were injected into the dorsal region near the thigh of each mouse. On day 1, 10 mice were randomly divided into two group (5 mice each group). 5x10^6^ T cells (50 µl) were incubated with 2 µg FcabCD3 or 2 µg human IgG along with 2000 IU IL2 (interleukin-2) for half an hour on ice, the mixture was then locally injected into the tumor site of each mouse. Repeated injection was continued on day 8 and on day 15 with 1 x10^7^ armed T cells, respectively. For tumor imaging, 2 mg D-luciferin (20 mg/ml in PBS, GoldBio Technology) was administrated i.p. (intra-peritoneally) into each mouse. Bioluminescent imaging (BLI) was measured using an IVIS Spectrum Imaging System (Caliper Life Science, Perkin Elmer Inc.). Mice were monitored for BLI on day 3, 10 and 17.

## Results

### Generation and characterization of FcabCD3

FcabCD3 was constructed as shown in Figure 1A . It contained a CD3 binder fused to an engineered Fcab fragment (Figure 1B and C). Puriﬁcation of both FcabCD3 and Fcab from the supernatants of transfected 293T cells yielded about 2 mg for each protein per liter culture.

On a calibrated Superdex 200^TM^ gel-filtration column, FcabCD3 eluted at 12.69 ml, equivalent to 158 kDa and Fcab eluted at 14.43 ml , equivalent to 75 kDa (Figure 2A). Purified proteins were analyzed on 12% SDS-PAGE. As shown in Figure 2B, FcabCD3 monomer migrated at an expected size of 60 kDa under reducing condition. Non-reduced FcabCD3 showed a molecular weight above 170 kDa on the SDS-PAGE gel. Meanwhile, under reducing condition, Fcab showed an expected size at 35 kDa and around 60 kDa under non-reducing condition. All above suggested that FcabCD3 existed as a homogenous dimer and it might have been glycosylated. 

### FcabCD3 Bound Specifically to HER2 Expressed on Tumor Cells

**Figure 3:**
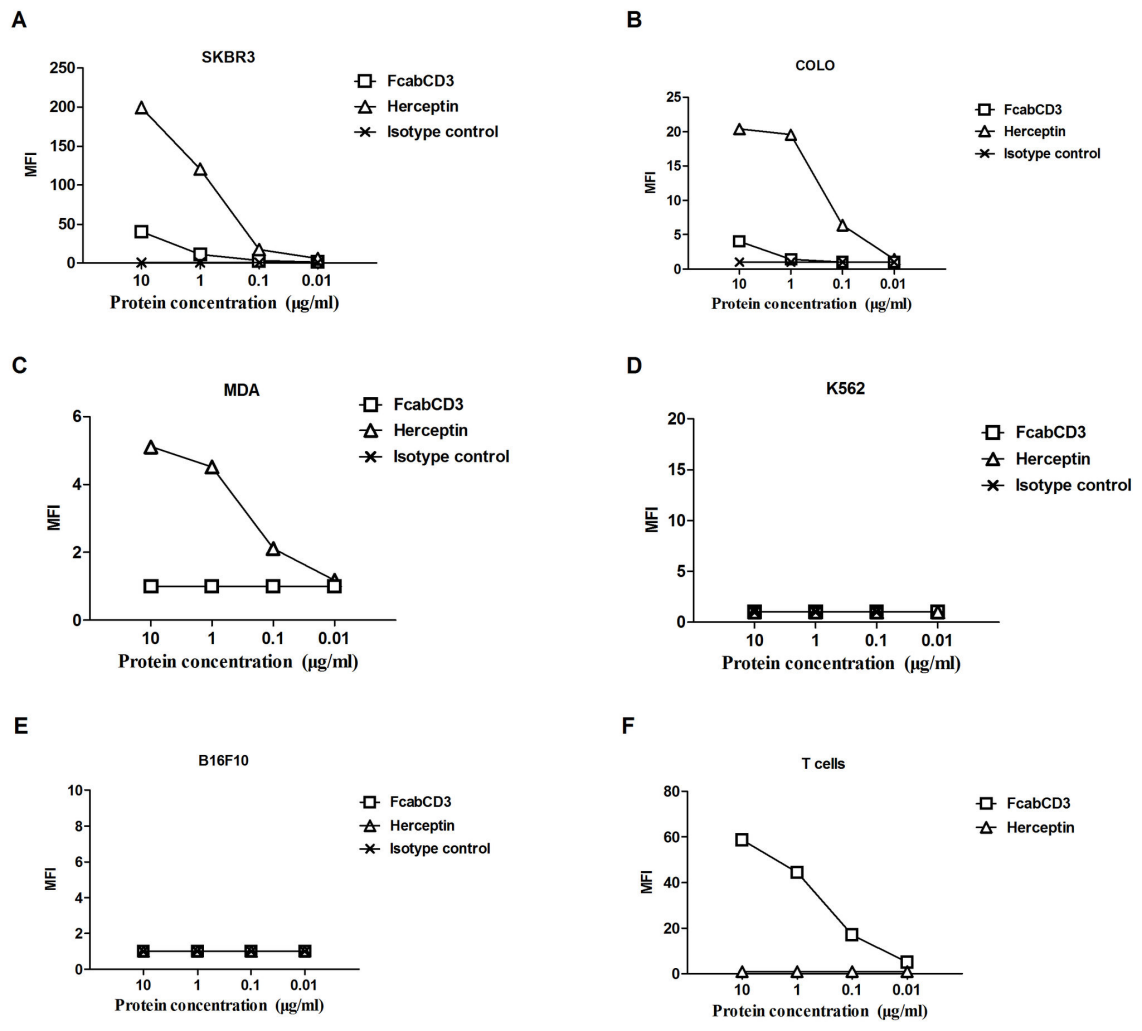
FACS analysis of FcabCD3 specificities. As described in Materials and Methods section, SKBR3, Colo205-luc, MDA-MB-231-luc, K562-luc, B16F10-luc cells and T cells were stained with serial dilutions of FcabCD3 and Herceptin. The MFI ratio was calculated by MFI of sample divided by MFI of isotype FITC control by FlowJo software.

Binding specificities of FcabCD3 to both CD3 and HER2 were analyzed by flow cytometry (Figure 3). According to FACS results and median fluorescent intensity (MFI), FcabCD3showed highest binding to HER2 on SKBR3 cells (MFI 40, Figure 3A) and moderately high on Colo205-luc cells (MFI 4, Figure 3B), but showed no binding on MDA-MB-231-luc cells (MFI 1, Figure 3C). As a positive control, Herceptin showed binding capabilities in all cell lines including MDA-MB-231-luc cells. 

Fcab has been reported to have a K_D_ value about 8.6 nM [16], whereas Herceptin had a much higher affinity (K_D_ value 0.1 nM) [19]. Therefore, MFI of FcabCD3 was found to be weaker than Herceptin. However, apart from its specificity to HER2, FcabCD3 also showed binding ability to CD3, thus confirming its bispecificity (Figure 3F). As expected no binding was detected on the K562 and B16F10 cell lines that expressed neither CD3 nor HER2 (Figure 3D and E).

### Redirected lysis of correspondent target cells mediated by FcabCD3 *in vitro*

T cells are known to play a pivotal role for killing tumor cells. The new bsAb FcabCD3 was tested to direct T cells to destroy HER2-positive tumor cells. The specific cytotoxicities at different E:T ratios with consistent antibody concentration and at different antibody concentrations with consistent E:T ratio were tested.

**Figure 4:**
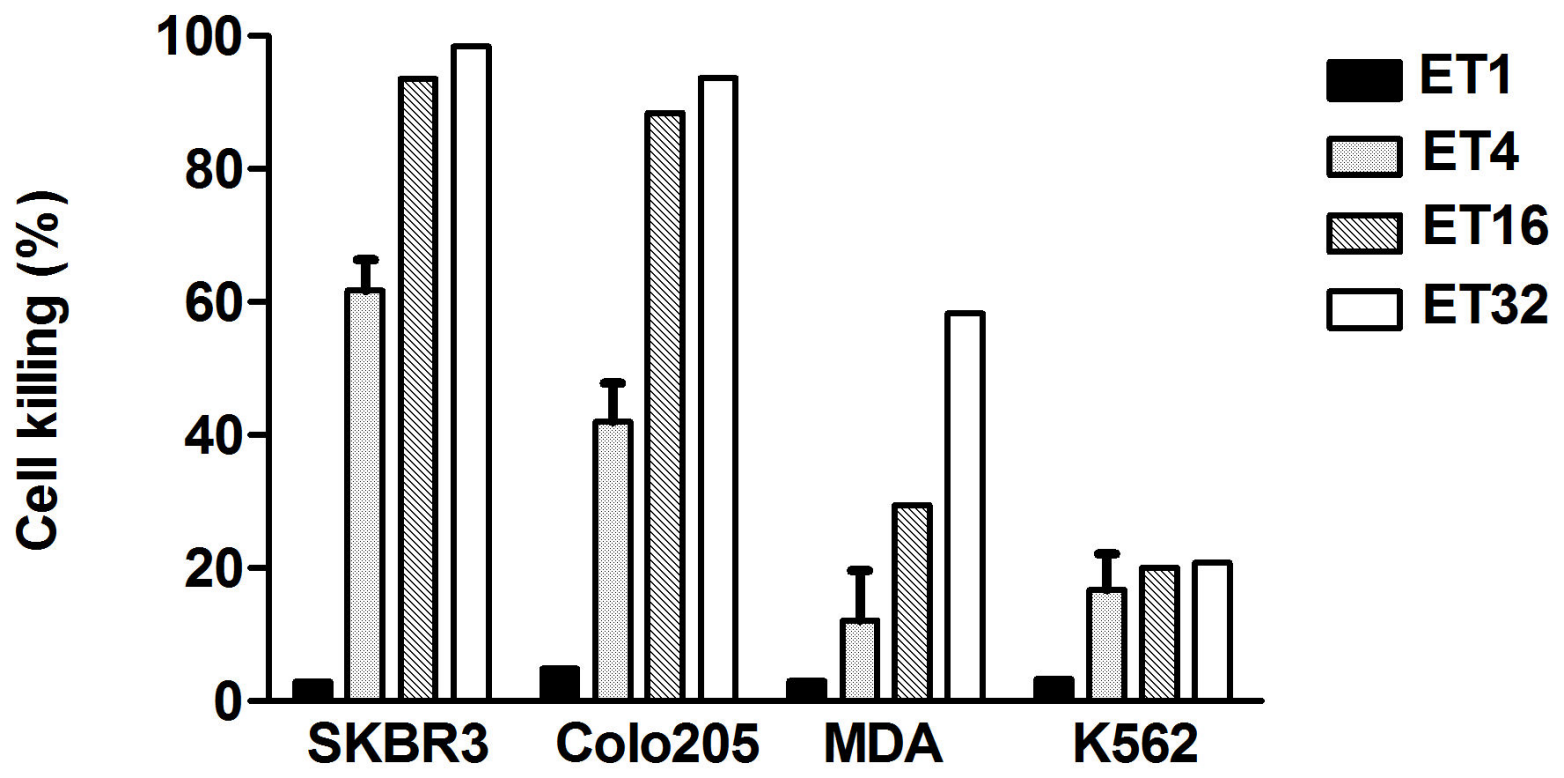
FcabCD3 directed killing of tumor cells by T cells at different E:T ratios. Different target cells were incubated with T cells at different E:T ratios. Incubation period for cytotoxicity for all cell lines was 20h except for SKBR3 48h. Cytotoxicity measured during coincubation without the addition of antibodies was subtracted from all samples.

First, specific cytotoxicity assay against SKBR3cell line (HER2 high expression) was evaluated at E:T ratios of 1, 4, 16 and 32 with 100 ng/ml FcabCD3 respectively. As shown in Figure 4, even at low E:T ratio (E:T=4), FcabCD3 armed T cells could kill more than 60% of SKBR3 tumor cells. The cytotoxicity assays to other cell lines expressing various levels of HER2 were performed based on bioluminescent killing assays. The result showed that FcabCD3 not only killed HER2-expressing Colo205-luc cells efficiently, but also showed cytotoxicity to MDA-MB-231-luc cells, a relatively low HER2 expressing cell line, as long as a high E:T ratio (E:T 32) was used (Figure 4). At different E:T ratios, no significant cytotoxicity was observed when HER2 negative cell line K562-luc was used as the target even at E:T 32. This demonstrated there was correlation between the killing efficiency and HER2 expression level on cell surface.

**Figure 5:**
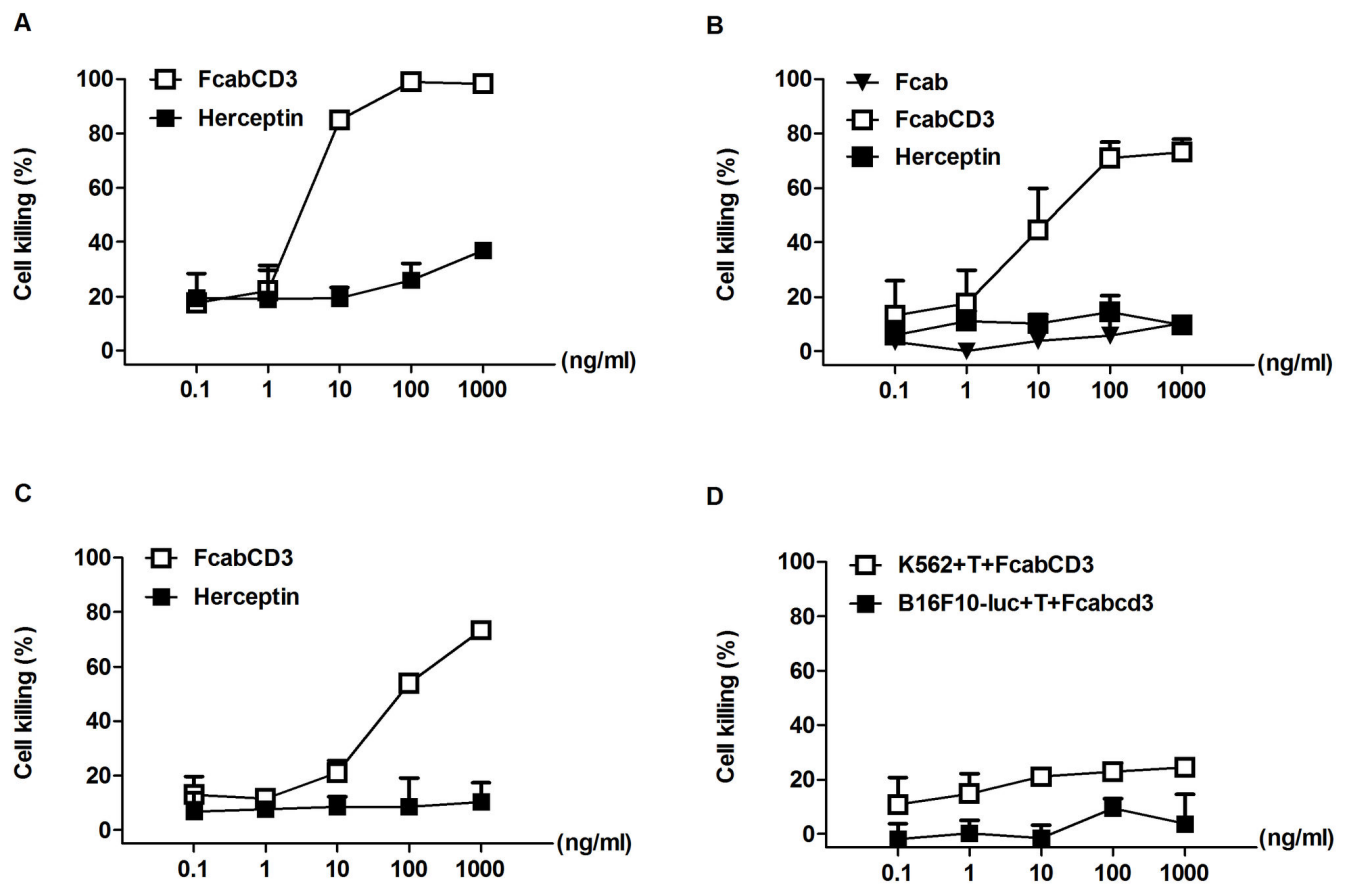
FcabCD3 directed killing of tumor cells by T cells at different bsAb concentrations. Dose-response analysis of serial dilutions of FcabCD3, Fcab and Herceptin on (A) SKBR3, (B) Colo205-luc, (C) MDA-MB-231-luc , (D) K562-luc and B16F10-luc cells at E:T10. Values are mean ±SD of triplicates. Statistical analysis for difference between speciﬁc tumor cell lysis was analyzed by one-way ANOVA method using SPSS software (SPSS, p<0.05).

With constant E:T ratio 10, cytotoxicities of serial dilutions of FcabCD3 were also evaluated. As shown in Figure 5A, as low as 10 ng/ml FcabCD3 could direct T cells to kill more than 80% of SKBR3 cells. At 1 µg/ml, all the SKBR3 cells were killed while Herceptin group showed only 40% lysis. Meanwhile, FcabCD3 showed efficient redirected T cell killing for Colo205-luc cells, whereas Fcab alone did not show sufficient killing activities as expected (Figure 5B). Since the parent Fcab fragment alone did not exhibit cytotoxicity, it indicated that killing was mainly directed by the CD3 positive T cells, not the natural killker (NK) or accessory cells. We also observed that Colo205-luc cells were insensitive to the Herceptin at 1 µg/ml (Figure 5B), even at as high as 10 µg/ml (data not shown). For HER2 low expressing cell line MDA-MB-231-luc, FcabCD3 armed T cells could efficiently lyse the target cells. While Herceptin failed to exert any cytotoxicity to MDA-MB-231-luc (Figure 5C). The concentration for 50% of maximal effect (EC_50_) of FcabCD3 for SKBR3, Colo205-luc , MDA-MB-231-luc cells was calculated to be 4.4 ng/ml, 9.4 ng/ml and 54.4 ng/ml respectively. All the values were calculated by GraphPad Prism 5 software at constant E:T ratio of 10.There was no obvious FcabCD3 directed cytotoxicity against K562-luc and B16F10-luc cells as expected (Figure 5D). All specific cytotoxicity was calculated by subtracting the background killing of T cells. Statistical signiﬁcance of differences in speciﬁc tumor cell lysis was analyzed by independent-samples t-test or one-way ANOVA method using SPSS software (SPSS, Chicago IL, USA, p<0.05).

**Figure 6:**
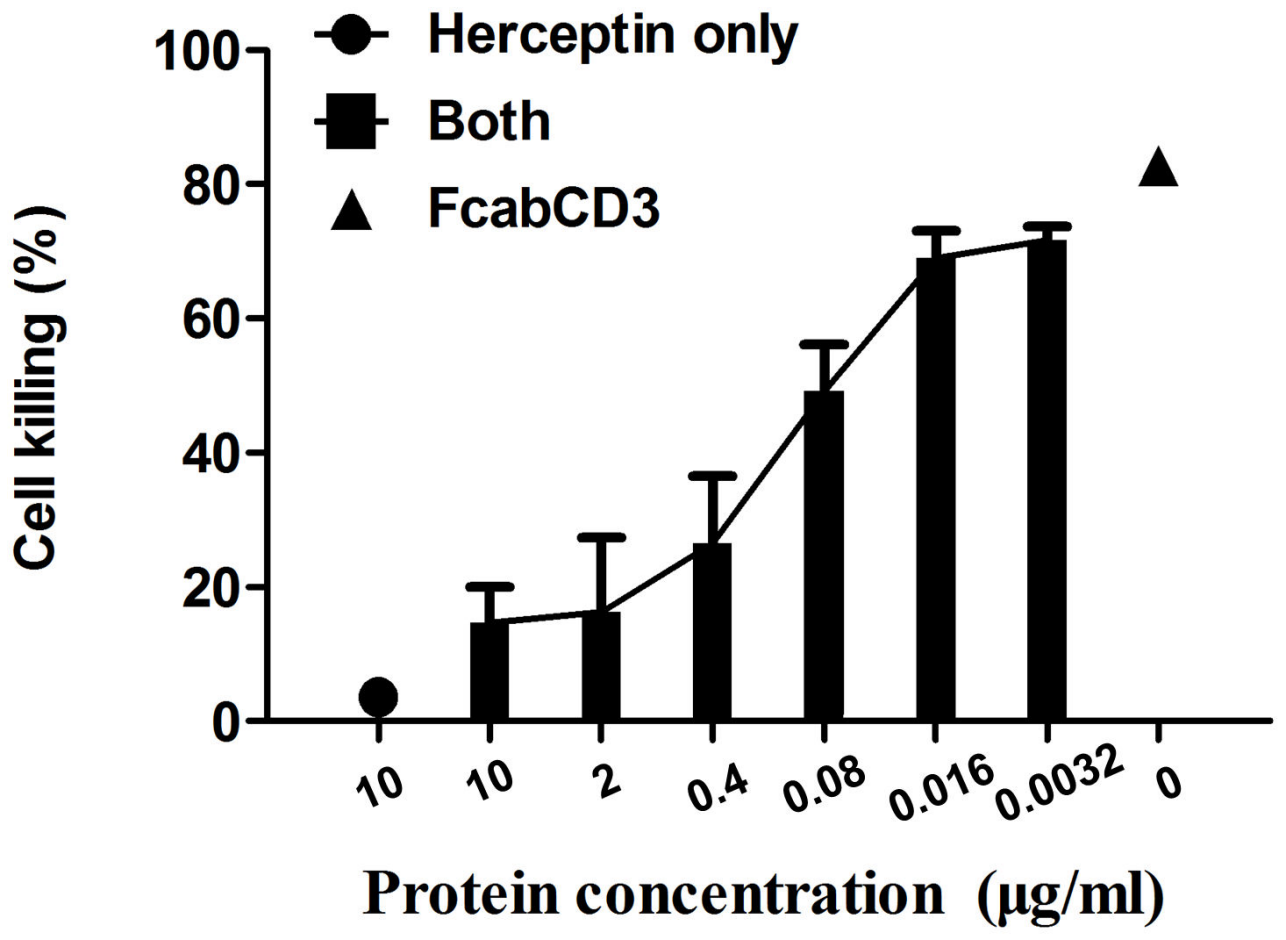
Inhibition of FcabCD3 killing by blocking of Herceptin. Killing was blocked by serial dilutions of Herceptin with FcabCD3 at a concentration of 100ng/ml. IC_50_ (0.076µg/ml) was calculated by GraphPad Prism 5 software.

Interestingly, the cytotoxicity of FcabCD3 to Colo205-luc cells could be blocked by Herceptin (Figure 6). This result suggested that FcabCD3 and Herceptin might recognize similar epitopes or there was steric hindrance to prevent the physical contact to HER2 by two antibodies simultaneously. Since the affinity of Herceptin to HER2 was much higher than FcabCD3, the blockage was almost 100% at a concentration of 10 µg/ml. The amount of Herceptin was titrated and the concentration for 50% of inhibition (IC_50_) (0.076 µg/ml) was calculated using GraphPad Prism 5.

### FcabCD3 armed T cells suppressed the growth of colon tumors in a xenograft model

**Figure 7:**
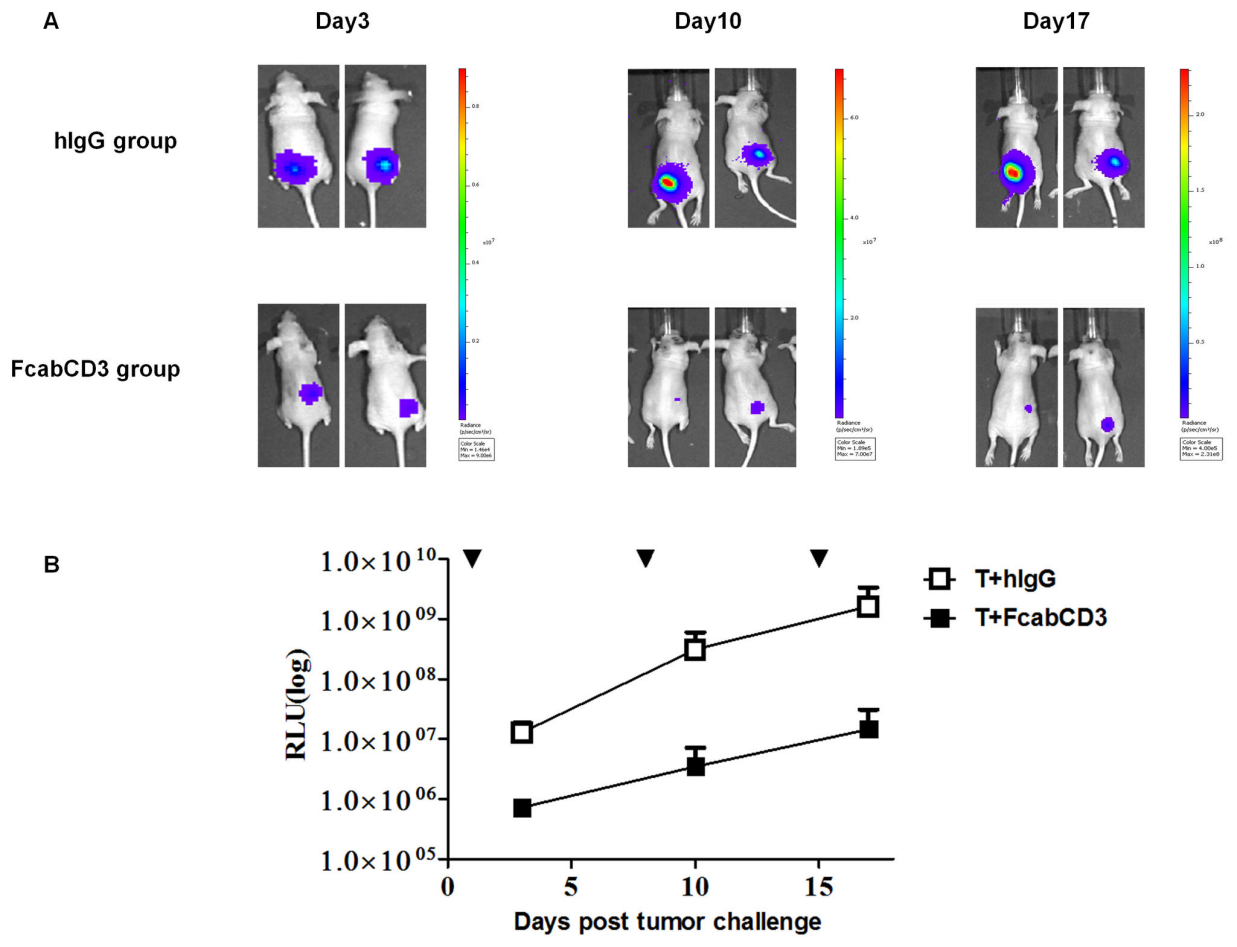
Anti-tumor effect of the FcabCD3 in a mouse tumor model. A, The tumor growth suppression by FcabCD3 on Colo205-luc cells was monitored by IVIS spectrum in nude mice. BALB/C strain nu/nu (n=10) mice were inoculated s.c. with Colo205-luc cells, and then randomly divided into two groups. On day1, 8 and 15, the mice were treated with FcabCD3 or human IgG (2µg per mouse) armed human T cells. On day 3, 10, 17, the tumor bioluminescence was measured;. B, The average value of relative light units (RLUs) was plotted against time (Days) in each group. Y-axis was in logarithmic scale.

For the evaluation of efficacy of the FcabCD3 to kill tumor cells *in vivo*, Colo205-luc cells were used and tumor growth was accurately measured by BLI [20]. Bioluminescence emitted from tumors on mice was detected using IVIS Spectrum Imaging System (Caliper Life Science, Perkin Elmer Inc.) and the photons emitted from Colo205-luc were quantified using Living Image software. As shown in Figure 7A, the tumors grew well after the engraftment of Colo205-luc cells in the human IgG control group, thus the T cells and IL2 alone had little effect on retardation of tumor growth. For the FcabCD3 group, signal of luciferase decreased on day 3, revealing significant tumor repression, and resulted in more than 2-fold reduction of BLI signal at the end of the treatment. The average relative light units (RLU) of each group (n=5) were shown in Figure 7B. Statistical significance of differences between two groups was calculated using SPSS software (Independent-samples t test, SPSS 11, p< 0.05). 

## Discussion

HER2 is an attractive target for breast cancer immunotherapy. Besides breast cancer, it is widely expressed on many human tumors including ovarian, bladder, salivary gland, endometrial, pancreatic and non-small-cell lung cancer (NSCLC) [21]. In this study, we fused an CD3 binder with Fcab which could bind to HER2 and mimic the full length antibody. Here, several cell lines containing luciferase as a reporter gene were used to demonstrate the sensitivities of different tumor cell lines to T cell lysis redirected by the bispecific antibody FcabCD3. Fu. et al. showed that there was an extremely good correlation between the cell numbers and the level of luciferase activity (R=0.99) [20]. Our result showed that FcabCD3 was highly efficient in killing HER2 positive tumor cell lines *in vitro* through a MHC non-restricted activation of T cells. The killing efficiency was well correlated with HER2 expression level in different cell lines. Furthermore, FcabCD3 armed T cells were shown to be effective in tumor inhibition *in vivo*.

Several promising bispecific antibodies targeting HER2 for recruiting effector cells were reported. Ertumaxomab (anti-HER2/anti-CD3) was produced by a quadroma cell line. As a new class of intact bsAb, Ertumaxomab could mobilize both T cells and FcγR type I/III+ cells (NK, macrophage, DC), it was highly effective in killing tumor cells expressing both high and low level of HER2 [22]. The major limitation of this format was the immunogenicity leading to HAMA(human anti mouse antibody) and HARA(human anti rat antibody). MDX-H210 (anti-FcγR1/anti-HER2) was prepared by chemically cross-linking of F(ab’) fragments of a mAb against FcγRI (CD64) with a mAb 520C9 against HER2. This chemically cross-linked bsAb in phase 1 clinical trial showed remarkable biological effects only when in combination with G-CSF [23]. Perhaps complicated manufacture and purification process would be its disadvantage. For the bsAb mentioned above, immunogenicity, yield and purity of the antibody were key obstacles impeding their application. Immunogenicity of bispecific fusion could be a concern when the repeated administration of the therapeutic antibody was necessary. Although both anti-CD3 binder and the engineered Fcab were de-immunized, whether the new construct might trigger undesired immune response remains to be fully elucidated and needs further investigation. FcabCD3 was expressed as a dimer in mammalian cells and purified to homogeneity by a one-step Protein A affinity chromatography. So FcabCD3 has a molecular weight less than an intact human IgG and is easy to produce in transiently transfected HEK-293T cells. Our result showed FcabCD3 had relatively high yield and purity, so it might be a better choice in application. A new format of bsAb named as T cell-engaging (BiTE) showed great potential in tumor therapy. Lutterbuese et al. [24] converted Trastuzumab (Herceptin) into BiTE antibody (anti-HER2/anti-CD3). The HER2-specific BiTE antibody showed potent redirected lysis of HER2-expressing target cell lines by peripheral human T cells. Due to its small molecular size (55 kDa) and short serum half life of several hours, BiTE was metabolized quickly and needed a pump to continuously deliver the antibody to maintain a steady-state serum levels [25]. In case of FcabCD3, there are several advantages to build an Fc fragment into a bsAb format. First, Fc domain can significantly increase bsAb’s half-life in blood, which prolongs therapeutic activity, owing to its interaction with the salvage neonatal Fc-receptor [9]. Similarly there would be a slower renal clearance for Fc incorporated antigen binding variable fragment comparing to scFv or BiTE bispecific format due to a larger sized molecules [10]. Second, Fc domain folds well and improves the stability of the partner molecule in a mammalian cell based expression system and bispecific fusion can be manufactured according to current available facility. Furthermore Fc region allows for a cost-effective purification with protein-A affinity chromatography during the production [26]. Third, Fc domain also enables bispecific molecules to bind to Fc-receptors (FcRs) found on immune cells to enhance their efficacy in immunotherapy [27]. Therefore, FcabCD3 would be a good candidate for the development of bispecific antibody format.

Morgan et al. [28]reported a case in which administration of 10^10^ T cells transduced with Chimeric Antigen Receptor (CARs) recognizing HER2 led to the death of a patient. Compared with an adoptive T cell therapy, the biggest difference of an antibody-based therapy is that antibodies are subjected to clearance by the body, therefore antibody-based therapy might be safer. Phase 1 clinical trial of tri-functional antibody Ertumaxomab [29] demonstrated that doses up to 100 µg was safe for patients and provided encouraging clinical benefits. Although the adverse events of the cytokines (IL6, IFN-γ, TNF-α) occurred, the concentrations of the released cytokines were much lower than adoptive T therapy method and the systemic inflammatory response syndrome was reversible. The dosage and affinity of bsAb should be balanced between the efficacy and adverse effects. FcabCD3 was highly effective in eliminating tumor cells both *in vitro* and *in vivo* at very low concentration. Although it is promising, the adverse events of FcabCD3 and the safety of the drug need further evaluation.

As a bsAb, FcabCD3 could direct T cells to target HER2 positive tumor both *in vitro* and *in vivo* while retaining the benefits of Fc fragment including longer half-life and better pharmacokinetics. These findings suggest FcabCD3 would be a favorable choice for HER2-based cancer immunotherapy. More importantly, the format provided a new strategy for treatment of cancer in the event when the expression of a target tumor antigen is low, eg, HER2 antigen. The format would be used to construct a novel class of bispecifics for tumor immunotherapy.
